# Receptor activator of nuclear factor kB ligand, osteoprotegerin, and risk of death following a breast cancer diagnosis: results from the EPIC cohort

**DOI:** 10.1186/s12885-018-4887-3

**Published:** 2018-10-22

**Authors:** Danja Sarink, Helena Schock, Theron Johnson, Jenny Chang-Claude, Kim Overvad, Anja Olsen, Anne Tjønneland, Patrick Arveux, Agnès Fournier, Marina Kvaskoff, Heiner Boeing, Anna Karakatsani, Antonia Trichopoulou, Carlo La Vecchia, Giovanna Masala, Claudia Agnoli, Salvatore Panico, Rosario Tumino, Carlotta Sacerdote, Carla H. van Gils, Petra H. M. Peeters, Elisabete Weiderpass, Antonio Agudo, Miguel Rodríguez-Barranco, José María Huerta, Eva Ardanaz, Leire Gil, Kay Tee Kaw, Julie A. Schmidt, Laure Dossus, Mathilde His, Dagfinn Aune, Elio Riboli, Rudolf Kaaks, Renée T. Fortner

**Affiliations:** 1 Division of Cancer Epidemiology, German Cancer Research Center (DKFZ), Im Neuenheimer Feld 280, 69120 Heidelberg, Germany; 2 Section for Epidemiology, Department of Public Health, Aarhus University, Aarhus, Denmark; 3 Danish Cancer Society Research Center, Copenhagen, Denmark; 4 Breast and Gynaecologic Cancer Registry of Côte d’Or, Georges-François Leclerc Comprehensive Cancer Care Centre, Dijon, France; 5 Université Paris-Saclay, Université Paris-Sud, UVSQ, CESP, INSERM, Villejuif, France; 6 Gustave Roussy, Villejuif, France; 7 Department of Epidemiology, German Institute of Human Nutrition Potsdam-Rehbruecke, Nuthetal, Germany; 8 Hellenic Health Foundation, Athens, Greece; 9 2nd Pulmonary Medicine Department, School of Medicine, National and Kapodistrian University of Athens, “ATTIKON” University Hospital, Haidari, Athens, Greece; 10 WHO Collaborating Center for Nutrition and Health, Unit of Nutritional Epidemiology and Nutrition in Public Health, Department of Hygiene, Epidemiology and Medical Statistics, School of Medicine, National and Kapodistrian University of Athens, Athens, Greece; 11 Department of Clinical Sciences and Community Health, University of Milan, Milan, Italy; 12 Cancer Risk Factors and Life-Style Epidemiology Unit, Cancer Research and Prevention Institute – ISP, Florence, Italy; 13 Epidemiology and Prevention Unit, Fondazione IRCCS Istituto Nazionale dei Tumori, Milan, Italy; 14 Dipartimento di Medicine Clinica e Chirurgia, Federico II University, Naples, Italy; 15 Cancer Registry and Histopathology Department, “Civic M.P.Arezzo” Hospital, Azienda Sanitaria Provinciale, Ragusa, Italy; 16 Unit of Cancer Epidemiology, Città della Salute e della Scienza University Hospital and Center for Cancer Prevention (CPO), Turin, Italy; 17 Department of Epidemiology, Julius Center for Health Sciences and Primary Care, University Medical Center Utrecht, Utrecht, the Netherlands; 18 MRC-PHE Centre for Environment and Health, Department of Epidemiology and Biostatistics, School of Public Health, Imperial College, London, UK; 19 Department of Community Medicine, Faculty of Health Sciences, University of Tromsø, The Arctic University of Norway, Tromsø, Norway; 20 Department of Research, Cancer Registry of Norway, Institute of Population-Based Cancer Research, Oslo, Norway; 21 Department of Medical Epidemiology and Biostatistics, Karolinska Institutet, Stockholm, Sweden; 22 Genetic Epidemiology Group, Folkhälsan Research Center, Helsinki, Finland; 23 Unit of Nutrition and Cancer, Cancer Epidemiology Research Program, Catalan Institute of Oncology-IDIBELL, L’Hospitalet de Llobregat, Barcelona, Spain; 24 Escuela Andaluza de Salud Pública. Instituto de Investigación Biosanitaria ibs.GRANADA, Hospitales Universitarios de Granada/Universidad de Granada, Granada, Spain; 25 CIBER de Epidemiología y Salud Pública (CIBERESP), Madrid, Spain; 26 Department of Epidemiology, Murcia Regional Health Council, IMIB-Arrixaca, Murcia, Spain; 27 Navarra Public Health Institute, Pamplona, Spain; 28 IdiSNA, Navarra Institute for Health Research, Pamplona, Spain; 29 Public Health Division of Gipuzkoa, Biodonostia Health Research Institute, San Sebastian, Spain; 30 Cancer Epidemiology Unit, University of Cambridge, Cambridge, UK; 31 Cancer Epidemiology Unit, Nuffield Department of Population Health, University of Oxford, Oxford, UK; 32 International Agency for Research on Cancer, Lyon, France; 33 Department of Epidemiology and Biostatistics, The School of Public Health, Imperial College London, London, UK

**Keywords:** Breast cancer, Reproductive, hormonal, and related factors, Epidemiology, Serum biomarkers of endogenous exposures

## Abstract

**Background:**

Receptor activator of nuclear factor kappa-B (RANK)-signaling is involved in tumor growth and spread in experimental models. Binding of RANK ligand (RANKL) to RANK activates signaling, which is inhibited by osteoprotegerin (OPG). We have previously shown that circulating soluble RANKL (sRANKL) and OPG are associated with breast cancer risk. Here we extend these findings to provide the first data on pre-diagnosis concentrations of sRANKL and OPG and risk of breast cancer-specific and overall mortality after a breast cancer diagnosis.

**Methods:**

Two thousand six pre- and postmenopausal women with incident invasive breast cancer (1620 (81%) with ER+ disease) participating in the European Prospective Investigation into Cancer and Nutrition (EPIC) cohort were followed-up for mortality. Pre-diagnosis concentrations of sRANKL and OPG were quantified in baseline serum samples using an enzyme-linked immunosorbent assay and electrochemiluminescent assay, respectively. Hazard ratios (HRs) and 95% confidence intervals (CIs) for breast cancer-specific and overall mortality were calculated using Cox proportional hazards regression models.

**Results:**

Especially in women with ER+ disease, higher circulating OPG concentrations were associated with higher risk of breast cancer-specific (quintile 5 vs 1 HR 1.77 [CI 1.03, 3.04]; p_trend_ 0.10) and overall mortality (q5 vs 1 HR 1.39 [CI 0.94, 2.05]; p_trend_ 0.02). sRANKL and the sRANKL/OPG ratio were not associated with mortality following a breast cancer diagnosis.

**Conclusions:**

High pre-diagnosis endogenous concentrations of OPG, the decoy receptor for RANKL, were associated with increased risk of death after a breast cancer diagnosis, especially in those with ER+ disease. These results need to be confirmed in well-characterized patient cohorts.

**Electronic supplementary material:**

The online version of this article (10.1186/s12885-018-4887-3) contains supplementary material, which is available to authorized users.

## Background

The RANK-axis consists of three tumor necrosis superfamily (TNF) members; receptor activator of nuclear factor kappa-B (RANK), its ligand (RANKL), and osteoprotegerin (OPG). Binding of RANKL to RANK promotes cell proliferation and, in experimental models, promotes primary mammary tumorigenesis and mammary stem cell expansion [1–4]. RANK-signaling is a mediator of progesterone-signaling and overexpression of RANK in mouse-mammary-tumor-virus models of hormone responsive breast cancer shows increased rates of hyperplasia and tumor development [1, 5]. OPG is the decoy receptor for RANKL and can downregulate RANK-signaling. OPG additionally serves as a decoy receptor for TNF related apoptosis inducing ligand (TRAIL) and may downregulate TRAIL-signaling, a process promoting cell death, especially in estrogen receptor (ER) negative breast cancer cells [6].

There has been increasing interest in the RANK-axis with respect to breast cancer risk and prognosis given the availability of a RANKL inhibitor, denosumab, which has been shown to reduce skeletal-related events in breast cancer patients with bone metastases [7] and may improve disease-free survival in postmenopausal breast cancer patients with ER and progesterone receptor (PR) positive disease [8]. We and others have recently shown that both sRANKL (soluble homotrimeric isoform of RANKL) and OPG concentrations in circulation may influence risk of breast cancer in humans [9–13]. Following our earlier investigations on pre-diagnosis sRANKL and OPG and breast cancer risk, the aim of this study was to investigate associations between pre-diagnosis concentrations of sRANKL and OPG and risk of death after a breast cancer diagnosis. Given the results reported to date, we hypothesized (1) higher sRANKL and lower OPG would be associated with higher risk of breast cancer-associated death among women with ER+ breast cancer; and, (2) a positive association between OPG and risk of breast cancer-associated death among women with ER- disease.

This study provides the first data on circulating RANK-axis members and breast cancer-specific mortality risk and the first data on differences in mortality risk by tumor hormone receptor status.

## Methods

### Study population: European Prospective Investigation into Cancer and Nutrition

The European Prospective Investigation into Cancer and Nutrition (EPIC) recruited more than 520,000 participants (367,993 women), aged predominantly 35–75 years, between 1992 and 2000 in ten European countries (Denmark, France, Germany, Greece, Italy, the Netherlands, Norway, Sweden, Spain, and the United Kingdom). Detailed dietary, reproductive, lifestyle, anthropometric, and medical history data were collected using standardized methods [14]. Incident cancer cases were identified through cancer registries in most countries; France, Germany, Greece, and the Naples (Italy) center conducted follow-up through review of health insurance records, contact with cancer and pathology registries, and/or direct contact with cohort members. Mortality data were obtained via active follow-up with participants and their next of kin in Germany and Greece, and via national and regional mortality registries in the remaining countries [16].

### Blood sample collection

A total of 64% (*n* = 235,607) of women provided a blood sample at baseline. Blood samples were collected according to standardized protocols. As independent studies on breast cancer were conducted by the Swedish centers, participants from these centers were not included in the current study. For all countries included in this study, except Denmark, half of the aliquots were stored locally and the other half centrally at the International Agency for Research on Cancer (IARC). The samples used in this study were stored at IARC under liquid nitrogen at − 196 °C, or locally at − 150 °C for Danish participants.

The EPIC study protocol was approved by ethical committees of all participating centers and all participants gave written informed consent. The protocol for the current study was approved by the ethical committees of the International Agency for Research on Cancer (IARC; project no. 12–42) and the University of Heidelberg (project no. S311/2014).

### Study design

**Fig. 1:**
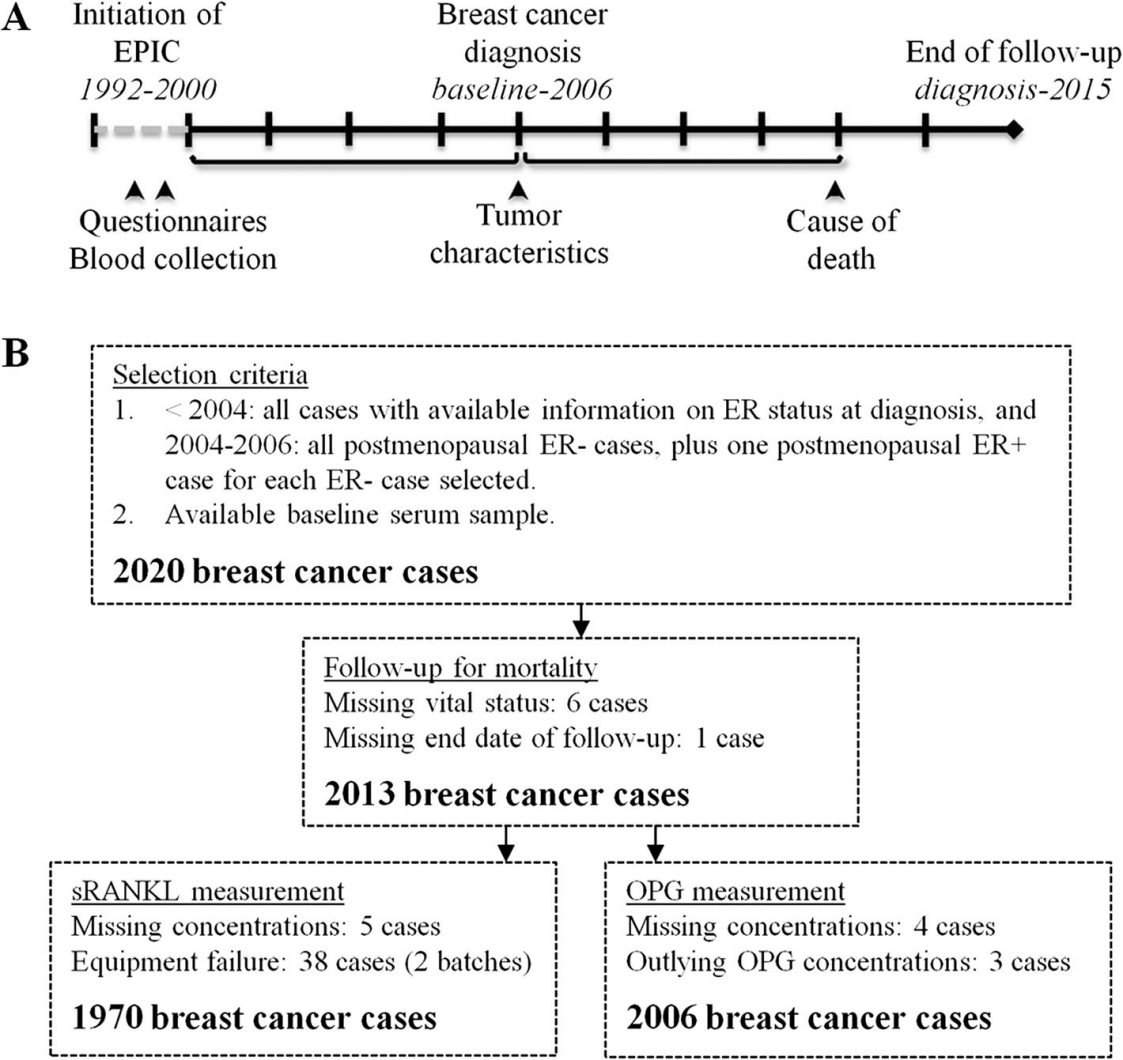
Study design and data collection. **a** Data were collected at three timepoints. Baseline blood samples were collected between 1992 and 2000. Breast cancer cases were diagnosed between baseline and initiation of the case-control study in 2006, and tumor characteristics at the time of breast cancer diagnosis were collected. Participants were followed-up for mortality from the time of breast cancer diagnosis to 2015. **b** 2020 breast cancer cases were initially selected for the case-control study. Of these, 1970 cases had complete information on sRANKL concentrations and follow-up for mortality, and 2006 had complete information on OPG concentrations and follow-up

The breast cancer cases in this study were part of a case-control study nested within the EPIC cohort. The study design and methods have been described previously [9, 12, 15]. Briefly, women diagnosed with a first invasive breast cancer between blood collection (ranging from 1992 to 2000 between centers) and completion of last follow-up for breast cancer incidence at the time the case-control study was initiated (ranging from 2003 to 2006 between centers) were included (Fig. 1a). The majority of EPIC participants were followed-up for breast cancer incidence and subsequent mortality via national or regional registries [16], and available information on tumor characteristics (e.g., hormone receptor subtype and stage at diagnosis) was collected where available. End of follow-up for mortality was defined as date of last complete follow-up for vital status, death, or emigration, and ranged from 2009 to 2015 between centers.

All cases with available serum sample and information on ER status of the tumor were eligible for the nested case-control study (Fig. 1b). From 2004, all postmenopausal ER- breast cancer cases were included, with one ER+ case randomly selected for every ER- case (matched on center). A total of 2020 breast cancer cases were initially available for the current analyses however, seven had no follow-up information after their breast cancer diagnosis and were excluded.

### Laboratory analyses

Pre-diagnosis sRANKL and OPG concentrations were analyzed at the Laboratory of the Division of Cancer Epidemiology at the German Cancer Research Center (DKFZ). Free serum sRANKL was quantified using an enzyme-linked immunosorbent assay (Biomedica, Austria), total serum OPG using an electrochemiluminescence assay (MesoScale Diagnostics, USA). All batches included the same serum quality control samples in duplicate to monitor inter-batch variation. Measurements and standard curves were done on a Victor system using Workout 2.5 software (Perkin Elmer) for sRANKL. The Quickplex SQ 120 Reader and Workbench 4.0.12 software (MesoScale Diagnostics) were used to measure OPG and create standard curves.

Of the 2013 breast cancer cases with complete follow-up data, four were missing OPG and 44 were missing sRANKL concentrations (38 cases, equipment failure and insufficient volume to re-assay; Fig. 1b). 152 cases (7.5%) with sRANKL values below the lower limit of detection (LLOD, 0.01 pmol/L) were set to half of the LLOD.

Inter-batch coefficients of variation were 1.2% for sRANKL and 16.6% for OPG. Intra-batch coefficients of variation were 14.4% for sRANKL, and 15.3% for OPG. Within-person reproducibility of sRANKL and OPG over one and 14 years observed in our study have been published previously [9, 12], Spearman correlation coefficients were *r* = 0.85 and *r* = 0.75 for OPG and *r* = 0.60 and *r* = 0.38 for sRANKL over one and 14 years, respectively.

### Statistical analyses

Pre-diagnosis sRANKL and OPG concentrations were log_2_ transformed to normalize the distributions, and to allow estimation of the effect of a doubling in concentrations. The ratio was calculated by dividing sRANKL concentrations by OPG concentrations; the ratio was then log_2_ transformed.

Outliers were evaluated using the extreme studentized deviate test [17]; three participants with outlying OPG concentrations (two with low, one with high OPG concentrations) were excluded from analyses (Fig. 1b). The final study population included 2006 breast cancer cases with OPG, 1970 with sRANKL, and 1965 cases with both.

We used Cox proportional hazards regression to estimate hazard ratios (HR) and 95% confidence intervals (CI) for risk of breast cancer-specific and all-cause mortality, using time since diagnosis as the time scale. sRANKL, OPG, and the sRANKL/OPG ratio were modeled as quintiles; tests for trend were calculated using continuous (log_2_) variables. The proportional hazards assumption was assessed using Schoenfeld residuals [18]. Based on our previous work on breast cancer risk showing significant heterogeneity by hormone receptor status [9, 12], and the oversampling of ER- cases after 2004, we decided a priori to evaluate associations for mortality both overall and by ER status. Similarly, confounders were selected a priori. Multivariable models were adjusted for body mass index (BMI; continuous), age at blood collection (continuous), age group at menarche (≤12, 13 and missing (as very few cases were missing information), 14, ≥15 years), age group at menopause (premenopausal, ≤48, 49–51, ≥52 years, missing), age group at first full term pregnancy (nulliparous, < 25, ≥25 years and missing), and breast cancer stage (localized, non-localized (including regional, distant, and unspecified metastatic sites), missing). Models were stratified by age at diagnosis (5-year age groups) and tumor ER status (negative or positive, in models among the whole population), as these variables violated the proportional hazards assumption. Additional adjustment for use of oral contraceptives or postmenopausal hormones at blood collection did not impact results (log2 HR < 10% change). Pre-diagnosis concentrations of sRANKL and OPG were weakly inversely correlated, with Spearman correlations of r = −0.25 in premenopausal and r = −0.32 in postmenopausal women.

Non-parametric restricted cubic splines were used to examine possible non-linearity, comparing models with linear and cubic terms to models with only the linear term [19]. There was no evidence of significant deviation from linearity (*p* >  0.11). We evaluated interaction between pre-diagnosis sRANKL and OPG concentrations and reproductive and lifestyle factors by comparing models with an interaction term to models without, using likelihood ratio tests. We observed significant interaction between OPG and BMI (*p* ≤ 0.04 in the whole population and in ER+ cases), and thus investigated associations between pre-diagnosis OPG and mortality after a breast cancer diagnosis in stratified models (BMI </≥ 25 kg/m^2^; i.e. non-overweight/overweight). We observed no significant interaction for the remaining factors (*p* ≥ 0.05), including menopausal status at blood collection, ages at blood collection, menarche, menopause, and first full term pregnancy, and postmenopausal hormone (PMH) use at blood collection. Similarly, there was no heterogeneity in associations by breast cancer stage at diagnosis (localized vs. non-localized). Information on breast cancer treatment was not available, thus we were not able to evaluate treatment-related factors as covariates or effect modifiers.

In addition to the ratio of pre-diagnosis sRANKL and OPG concentrations we evaluated mutually adjusted models (i.e. sRANKL models additionally adjusted for log_2_ OPG concentrations and vice versa) and a cross-classification of pre-diagnosis sRANKL and OPG at the median concentration (as OPG may inhibit sRANKL signaling, those with sRANKL concentrations ≤ median and OPG concentrations > median were chosen as the reference group). We further conducted a sensitivity analysis excluding those diagnosed within two years of blood collection to address potential reverse causation.

All statistical tests were two-tailed and considered significant at *p* < 0.05. Statistical analyses were conducted using SAS 9.4 (SAS Institute Inc., Cary, NC, USA).

## Results

### Population characteristics

Among 2006 breast cancer cases in whom OPG concentrations were available, the median age at blood collection was 56.6 (range: 26.7, 75.5) years; 1543 (76.9%) of breast cancer cases were postmenopausal and of these, 48.8% were using PMH at blood collection (Table 1). The majority (86%) of cases had at least one full term pregnancy, with a median age at first full term pregnancy of 25 (16.0, 44.0) years. sRANKL and OPG concentrations were measured in blood samples collected a median of 4.7 (0.02, 11.7) years before breast cancer diagnosis. The median age at diagnosis was 60.9 (35.2, 83.6) years and the majority of cases (*n* = 1620, 80.8%) were diagnosed with ER+ breast cancer. The median time between diagnosis and end of follow-up was 10.9 (0.05, 19.1) years; the median survival time between diagnosis and death was 6.5 (0.1, 18.5) years among those who died of any cause and 5.0 (0.8, 15.2) years among those who died of breast cancer. A total of 421 deaths, including 250 breast cancer deaths, occurred over 21,253 person years of follow-up (Table 1). Compared to those diagnosed with ER- breast cancer, those diagnosed with ER+ disease where slightly older at blood collection and diagnosis, more likely to also have PR+ disease, and had a longer survival time. Though sRANKL concentrations were available for a smaller population than OPG concentrations (*n* = 1970 cases for sRANKL and *n* = 1965 for the sRANKL/OPG ratio), population characteristics were very similar (data not shown).

**Table 1:** Characteristics of the full study population and ER+ and ER- subgroups

	Full population	ER+ cases	ER- cases
*n* ^*a*^	2006	1620	386
*Baseline characteristics, median (range), or n (%))*			
Age at blood collection, years	56.6 (26.7, 75.5)	56.8 (33.4, 75.5)	54.9 (26.7, 72.1)
Age at menarche, years*	13.0 (8.0, 20.0)	13.0 (8.0, 20.0)	13.0 (9.0, 20.0)
Menopausal status at blood collection			
Premenopausal	463 (23%)	352 (22%)	111 (29%)
Postmenopausal	1543 (77%)	1268 (78%)	275 (71%)
PMH use at blood collection^b^	753 (49%)	623 (49%)	130 (47%)
Age at menopause, years^b*^	50.0 (27.0, 63.0)	50.0 (27.0, 63.0)	49.0 (30.0, 59.0)
Full term pregnancy, ever*	1693 (86%)	1356 (85%)	337 (89%)
Age at first term pregnancy, years^c*^	25.0 (16.0, 44.0)	25.0 (16.0, 44.0)	24.0 (16.0, 38.0)
BMI, kg/m^2^	24.4 (13.8, 49.1)	24.5 (13.8, 49.1)	24.0 (16.6, 45.4)
sRANKL concentrations (pmol/L)^a^	0.11 (0.005, 1.67)	0.11 (0.005, 1.67)	0.11 (0.005, 0.58)
OPG concentrations (pmol/L)	9.86 (2.96, 31.97)	9.95 (2.96, 31.97)	9.59 (3.14, 21.38)
sRANKL/OPG ratio ^a^	0.01 (0.0002, 0.17)	0.01 (0.0002, 0.17)	0.01 (0.0002, 0.09)
*Breast cancer characteristics*			
Age at diagnosis, years	60.9 (35.2, 83.6)	61.3 (37.1, 83.6)	59.1 (35.2, 80.6)
Time between blood collection and diagnosis, years	4.7 (0.02, 11.7)	4.8 (0.02, 12.0)	4.5 (0.04, 11.5)
Progesterone receptor subtype at diagnosis*			
PR+	984 (67%)	926 (80%)	58 (18%)
PR-	494 (33%)	236 (20%)	258 (82%)
Breast cancer stage at diagnosis*			
Localized	1089 (68%)	877 (68%)	212 (66%)
Non-localized^d^	524 (32%)	415 (32%)	109 (34%)
*Deaths & follow-up*			
Number of deaths of any cause	421 (21.0%)	310 (19%)	111 (29%)
Number of breast cancer deaths	250 (12.5%)	165 (10%)	85 (22%)
Duration of follow-up, years^e^	10.9 (0.05, 19.1)	11.0 (0.05, 19.1)	10.3 (0.08, 18.3)
Overall survival time, years^f^	6.5 (0.08, 18.5)	7.2 (0.14, 18.5)	4.1 (0.08, 15.2)
Breast cancer-specific survival time, years^g^	5.0 (0.8, 15.2)	6.4 (0.7, 14.8)	3.4 (0.08, 15.2)

^a^*n* = 1970 for sRANKL analyses and *n* = 1965 for sRANKL/OPG ratio analyses, comparable distribution of baseline characteristics; ^b^ Among postmenopausal women; ^c^ among women with at least one completed term pregnancy; ^d^ non-localized breast cancer includes regional (*n* = 401), distant (*n* = 15), and unspecified (*n* = 108) metastatic sites; ^e^ time between diagnosis and end of follow-up; ^f^ Time between diagnosis and death, among those who died of any cause; ^g^ Time between diagnosis and death, among those who died of breast cancer. * Missing data: Age at menarche: 36 cases (1.8%); age at menopause: 477 cases (31%); ever FTP: 30 cases (1.5%); age at first FTP: 7 cases (0.4%); PR status: 528 cases (26%); breast cancer stage: 393 cases (19.6%)

### OPG concentrations

Higher pre-diagnosis OPG concentrations were associated with an increased risk of breast cancer-specific mortality among women with ER+ disease (quintile (q)5 vs. q1 HR 1.77 [CI 1.03, 3.04]; p_trend_ 0.10) (Table 2). Additional adjustment for pre-diagnosis sRANKL concentrations strengthened this association (q5 vs q1 HR 2.02 [1.15, 3.54]; p_trend_ 0.07). For all-cause mortality, higher pre-diagnosis concentrations of OPG were associated with a suggestive increased risk of mortality in all cases (q5 vs. q1 HR 1.25 [CI 0.90, 1.73]; p_trend_ 0.02) and in ER+ cases (q5 vs. q1 HR 1.39 [CI 0.94, 2.05); p_trend_ 0.02). Though the *p*-value for linear trend was attenuated, additional adjustment for pre-diagnosis sRANKL concentrations did not substantially impact risk estimates (Table 2). We did not observe heterogeneity by ER status at diagnosis (p_het_ 0.58); however, OPG was not associated with mortality risk in those diagnosed with ER- breast cancer.

**Table 2:** Circulating concentrations of OPG and risk of death following a breast cancer diagnosis, by ER subtype

*Cut points (pmol/L)*	Quintiles					p_trend_^a^	HR_log2_	p_het_^b^
	1	2	3	4	5			
	≤ 7.80	7.80–9.18	9.18–10.54	10.54–12.38	> 12.38			
Breast cancer-specific death								
All breast cancer cases								
*Died/total*	51/402	48/401	44/401	55/401	52/401		250/2006	
*HR (95% CI)*	Ref.	1.04 (0.70, 1.57)	0.96 (0.63, 1.48)	1.33 (0.88, 2.00)	1.37 (0.90, 2.09)	0.13	1.30 (0.93, 1.80)	0.58
*sRANKL adjusted* *HR (95% CI)*^*c*^	Ref.	1.08 (0.72, 1.63)	1.02 (0.66, 1.57)	1.32 (0.86, 2.01)	1.45 (0.93, 2.27)	0.12	1.32 (0.93, 1.88)	0.55
ER+ breast cancer cases								
*Died/total*	31/318	35/321	27/322	35/328	37/331		165/1620	
*HR (95% CI)*	Ref.	1.34 (0.81, 2.20)	1.08 (0.62, 1.85)	1.47 (0.88, 2.46)	1.77 (1.03, 3.04)	0.10	1.43 (0.94, 2.17)	
*sRANKL adjusted* *HR (95% CI)*^*c*^	Ref.	1.44 (0.87, 2.39)	1.17 (0.67, 2.03)	1.52 (0.89, 2.59)	2.02 (1.15, 3.54)	0.07	1.52 (0.97, 2.36)	
ER- breast cancer cases								
*Died/total*	20/84	13/80	17/79	20/73	15/70		85/386	
*HR (95% CI)*	Ref.	0.71 (0.34, 1.51)	0.77 (0.38, 1.57)	1.27 (0.63, 2.54)	0.91 (0.44, 1.89)	0.60	1.16 (0.68, 1.99)	
*sRANKL adjusted* *HR (95% CI)*^*c*^	Ref.	0.70 (0.32, 1.52)	0.80 (0.38, 1.68)	1.20 (0.58, 2.52)	0.86 (0.38, 1.93)	0.72	1.12 (0.62, 2.02)	
Death of any cause								
All breast cancer cases								
*Alive/ total*	77/402	71/401	71/401	93/401	109/401		421/2006	
*HR (95% CI)*	Ref.	0.88 (0.64, 1.23)	0.83 (0.59, 1.17)	1.10 (0.80, 1.52)	1.25 (0.90, 1.73)	0.02	1.37 (1.05, 1.78)	0.66
*sRANKL adjusted* *HR (95% CI)*^*c*^	Ref.	0.90 (0.65, 1.26)	0.83 (0.59, 1.17)	1.04 (0.75, 1.45)	1.20 (0.85, 1.69)	0.07	1.29 (0.98, 1.70)	0.66
ER+ breast cancer cases								
*Died/total*	53/318	54/321	47/322	71/328	84/330		310/1620	
*HR (95% CI)*	Ref.	1.00 (0.68, 1.48)	0.83 (0.55, 1.26)	1.20 (0.82, 1.75)	1.39 (0.94, 2.05)	0.02	1.44 (1.06, 1.96)	
*sRANKL adjusted* *HR (95% CI)*^*c*^	Ref.	1.04 (0.70, 1.54)	0.85 (0.56, 1.29)	1.17 (0.79, 1.72)	1.38 (0.92, 2.08)	0.05	1.39 (1.00, 1.92)	
ER- breast cancer cases								
*Died/total*	24/84	17/80	24/79	22/73	24/70		111/386	
*HR (95% CI)*	Ref.	0.70 (0.36, 1.35)	0.80 (0.43, 1.49)	1.02 (0.54, 1.93)	1.04 (0.55, 1.94)	0.34	1.27 (0.78, 2.08)	
*sRANKL adjusted* *HR (95% CI)*^*c*^	Ref.	0.68 (0.34, 1.35)	0.74 (0.38, 1.42)	0.90 (0.46, 1.75)	0.86 (0.43, 1.32)	0.75	1.09 (0.63, 1.89)	

All models adjusted for breast cancer stage (localized, non-localized, missing), BMI (kg/m^2^), age at blood collection (years), and age groups at menarche (≤11, 12, 13 and missing, 14, ≥15 years), menopause (premenopausal ≤ 48, 49–51, ≥52 years, missing), and first full term pregnancy (nulliparous, < 25 years, ≥25 years and missing); stratifying by age groups at diagnosis (5 year groups) and models in all cases by ER status of the tumor

^a^p_trend_ based on log2-transformed OPG concentrations; ^b^ p_heterogeneity_ comparing model without to model with interaction term for OPG and ER status using log likelihood ratio tests; ^c^ additionally adjusting for sRANKL concentrations (42 observations missing sRANKL concentrations; analyses include 245 breast cancer deaths, 412 deaths of any cause)

Stratifying by BMI at blood collection (p_int_ ≤ 0.04 in models for OPG among all cases and ER+ cases) showed that pre-diagnosis OPG concentrations were not associated with risk of death after a breast cancer diagnosis in those with a high BMI (≥ 25 kg/m^2^) at blood collection (Additional file 1: Table S1). Among those with a lower BMI (< 25 kg/m^2^), high pre-diagnosis concentrations of OPG were strongly associated with an increased risk of both breast cancer-specific and all-cause mortality, especially among those with ER+ disease (e.g. breast cancer mortality in ER+ cases q5 vs q1 HR 2.80 [1.30, 6.02]; p_trend_ 0.01, additionally adjusted for pre-diagnosis sRANKL concentrations HR 3.19 [1.46, 6.97]; p_trend_ 0.005).

### sRANKL concentrations

Pre-diagnosis sRANKL concentrations were not associated with breast cancer-specific or all-cause mortality (e.g. breast cancer-specific mortality, in the full population: q5 vs q1 HR 0.99 [0.66, 1.47]; p_trend_ 0.84; Table 3). Results were unchanged by additional adjustment for pre-diagnosis OPG concentrations.

**Table 3:** Circulating concentrations of sRANKL and risk of death following a breast cancer diagnosis, by ER subtype

*Cut points (pmol/L)*	Quintiles					p_trend_^a^	HR_log2_	p_het_^b^
	1	2	3	4	5			
	≤ 0.04	0.04–0.09	0.09–0.14	0.14–0.21	> 0.21			
Breast cancer-specific death								
All breast cancer cases								
*Died/total*	54/421	46/443	49/374	41/363	56/369		246/1970	
*HR (95% CI)*	Ref.	0.71 (0.47, 1.06)	0.98 (0.66, 1.45)	0.86 (0.57, 1.30)	0.99 (0.66, 1.47)	0.84	0.99 (0.92, 1.07)	0.31
*OPG adjusted* *HR (95% CI)*^*c*^	Ref.	0.76 (0.50, 1.14)	1.05 (0.70, 1.57)	0.94 (0.61, 1.44)	1.12 (0.74, 1.71)	0.70	1.02 (0.94, 1.11)	0.43
ER+ breast cancer cases								
*Died/total*	34/337	32/360	29/300	28/297	39/298		162/1592	
*HR (95% CI)*	Ref.	0.84 (0.51, 1.37)	0.98 (0.59, 1.63)	0.91 (0.54, 1.51)	1.08 (0.66, 1.77)	0.81	1.01 (0.92, 1.12)	
*OPG adjusted* *HR (95% CI)*^*c*^	Ref.	0.87 (0.53, 1.43)	1.05 (0.63, 1.74)	1.01 (0.60, 1.71)	1.24 (0.74, 2.08)	0.46	1.04 (0.94, 1.15)	
ER- breast cancer cases								
*Died/total*	20/84	14/83	20/74	13/66	17/71		84/378	
*HR (95% CI)*	Ref.	0.49 (0.23, 1.02)	1.05 (0.54, 2.05)	0.77 (0.37, 1.63)	0.76 (0.37, 1.55)	0.55	0.96 (0.84, 1.10)	
*OPG adjusted* *HR (95% CI)*^*c*^	Ref.	0.53 (0.25, 1.14)	1.14 (0.56, 2.29)	0.84 (0.39, 1.83)	0.85 (0.39, 1.86)	0.82	0.98 (0.84, 1.15)	
Death of any cause								
All breast cancer cases								
*Died/total*	103/421	91/443	73/374	68/363	79/369		414/1970	
*HR (95% CI)*	Ref.	0.80 (0.60, 1.07)	0.79 (0.58, 1.08)	0.80 (0.58, 1.09)	0.93 (0.68, 1.27)	0.10	0.95 (0.90, 1.01)	0.20
*OPG adjusted* *HR (95% CI)*^*c*^	Ref.	0.84 (0.63, 1.13)	0.85 (0.62, 1.16)	0.87 (0.63, 1.20)	1.04 (0.75, 1.45)	0.34	0.97 (0.91, 1.03)	0.31
ER+ breast cancer cases								
*Died/total*	75/337	69/360	50/300	50/297	61/298		305/1592	
*HR (95% CI)*	Ref.	0.85 (0.61, 1.18)	0.76 (0.53, 1.10)	0.76 (0.53, 1.10)	1.00 (0.70, 1.44)	0.31	0.97 (0.90, 1.03)	
*OPG adjusted* *HR (95% CI)*^*c*^	Ref.	0.87 (0.62, 1.22)	0.80 (0.55, 1.16)	0.84 (0.58, 1.22)	1.13 (0.78, 1.66)	0.66	0.98 (0.92, 1.06)	
ER- breast cancer cases								
*Died/total*	28/84	22/83	23/74	18/66	18/71		109/378	
*HR (95% CI)*	Ref.	0.62 (0.34, 1.15)	0.91 (0.50, 1.64)	0.86 (0.45, 1.61)	0.67 (0.35, 1.27)	0.11	0.91 (0.81, 1.02)	
*OPG adjusted* *HR (95% CI)*^*c*^	Ref.	0.69 (0.36, 1.30)	0.99 (0.53, 1.86)	0.94 (0.48, 1.82)	0.76 (0.37, 1.53)	0.24	0.92 (0.81, 1.06)	

All models adjusted for breast cancer stage (localized, non-localized, missing), BMI (kg/m^2^), age at blood collection (years), and age groups at menarche (≤11, 12, 13 and missing, 14, ≥15 years), menopause (premenopausal ≤ 48, 49–51, ≥52 years, missing), and first full term pregnancy (nulliparous, < 25 years, ≥25 years and missing); stratifying by age groups at diagnosis (5 year groups) and models in all cases by ER status of the tumor

^a^p_trend_ based on log2-transformed sRANKL concentrations; ^b^ p_heterogeneity_ comparing model without to model with interaction term for sRANKL and ER status using log likelihood ratio tests; ^c^ additionally adjusting for OPG concentrations (five observations missing OPG concentrations; analyses include 245 breast cancer deaths, 412 deaths of any cause)

### sRANKL/OPG ratio and cross-classification

A higher ratio between pre-diagnosis sRANKL and OPG concentrations was not associated with breast cancer-specific or overall mortality risk **(**Additional file 1: Table S2). We observed no associations between cross-classified pre-diagnosis sRANKL/OPG and breast cancer-specific mortality (Additional file 1: Table S3). In line with results for OPG, all-cause mortality risk was lower in ER+ cases with both pre-diagnosis sRANKL and OPG values below the median (HR 0.63 [CI 0.44, 0.92]), relative to those with low sRANKL and high OPG concentrations. Results for breast cancer-specific mortality were of similar magnitude, though not significant (HR 0.69 [0.42–1.15]).

### Sensitivity analyses

Information on PR status was available for 1478 (74%) of cases; stratification by both ER and PR status (i.e. ER + PR+ and ER-PR-) did not materially affect results (data not shown). Excluding those diagnosed with breast cancer within two years of blood collection did not impact results for pre-diagnosis sRANKL or the pre-diagnosis sRANKL/OPG ratio, but did attenuate associations between pre-diagnosis OPG concentrations and risk death (e.g. ER+ cases: all-cause mortality q5 vs q1 HR 1.24 [0.79–1.93]; p_trend_ 0.13 and breast cancer-specific q5 vs q1 HR 1.64 [0.89–3.05]; p_trend_ 0.23). There was no heterogeneity in associations by breast cancer stage at diagnosis (breast cancer-specific mortality p_het_ ≥ 0.18; all-cause mortality p_het_ ≥ 0.43), and we observed no heterogeneity in associations by menopausal status at blood collection for sRANKL and sRANKL/OPG (p_het_ ≥ 0.44). For OPG, we observed no heterogeneity in associations by menopausal status at blood collection for breast-cancer specific mortality (P_het_ ≥ 0.14), and suggestive heterogeneity in models for all-cause mortality (p_het_ = 0.05 in the full population). In analyses stratified by menopausal status at blood collection associations between OPG and all-cause mortality among postmenopausal women were similar to those in the full population (e.g. q5 vs. q1 HR 1.35 [CI 0.93, 1.98]; p_trend_ 0.004). In the smaller group of women premenopausal at blood collection (*n* = 75 deaths and 60 breast cancer deaths), log2 OPG concentrations were not associated with breast cancer risk.

## Discussion

In this large-scale prospective study, high pre-diagnosis concentrations of OPG were associated with an increased risk of death after an ER+ breast cancer diagnosis, especially among women with BMI less than 25 kg/m^2^. Pre-diagnosis sRANKL concentrations and the sRANKL/OPG ratio were not associated with mortality following a breast cancer diagnosis.

Experimental data in mouse models show RANKL blockade using OPG-Fc reduced formation of breast cancer metastases [20–22]. In studies in breast cancer patients, most reported either no or an inverse association between tumor RANKL or OPG expression and risk of death [23–27], recurrence [23–25, 27, 28], and metastasis [25, 29, 30], though this was not observed in all studies [31]. Our observation of higher risk of mortality following a breast cancer diagnosis in women with high circulating concentrations of OPG runs counter to these findings on expression in breast cancer tissue.

Few prior studies have evaluated circulating concentrations of sRANKL, OPG, and prognosis-related factors or mortality in breast cancer patients. Vik et al. evaluated cancer risk and mortality in the Trømso study and found no association between serum OPG concentrations measured a median of 13.5 years before diagnosis and cancer-related mortality in women [11]. However, there were too few cases to evaluate breast cancer-specific mortality or associations by hormone receptor status (76 incident breast cancer and 7 breast cancer deaths). Mountzios et al. found higher concentrations of sRANKL and OPG, as well as a higher sRANKL/OPG ratio, in 30 breast cancer patients with at least one recently diagnosed osteolytic or osteoblastic bone lesion compared to 22 healthy controls [32]. OPG was additionally found to be higher in those with more skeletal metastases (6–10 and ≥ 10 lesions compared to < 6 lesions). In contrast, Mercatali et al. found lower sRANKL and OPG concentrations in 54 breast cancer patients who underwent surgery and had bone metastases compared to both 30 healthy controls and 49 breast cancer patients who had ‘no evidence of disease’ after surgery [33]. Yao et al. evaluated correlates of sRANKL and OPG concentrations in 2401 breast cancer cases with serum samples collected median 73 days following breast cancer diagnosis. This study observed no association between sRANKL, OPG, or the sRANKL/OPG ratio and ER, PR, and HER2 status, and somewhat higher OPG concentrations among women diagnosed with stage IV disease [34]. In the same study, both RANKL and OPG were associated with age at diagnosis (i.e. time at which blood was collected). This is in line with the current study, where age at blood collection correlates moderately with OPG (*r* = 0.41) concentrations, though only weakly with sRANKL concentrations (*r* = − 0.15). We observed no difference in log-transformed sRANKL and OPG concentrations, or the sRANKL/OPG ratio by ER and/or PR status (negative vs positive; *p* ≥ 0.11) or by breast cancer stage at diagnosis (localized vs non-localized; *p* ≥ 0.23), nor did we observe any interaction by cancer stage at diagnosis in our Cox regression models (localized vs non-localized (including regional, distant, and unspecified metastatic sites) breast cancer-specific mortality p_het_ ≥ 0.18; all-cause mortality p_het_ ≥ 0.43). In a sensitivity analysis excluding 15 cases with distant metastases, associations with mortality were somewhat weakened.

The RANK-axis may be particularly relevant in BRCA mutation carriers [35]. In the current study, we were unable to restrict our analyses to a high-risk population. Information on BRCA mutation status was not available and information on family history of breast cancer is limited; 714 cases (40%) have any information available and of these, only 87 (12%) have a positively family history. We observed a positive association between higher pre-diagnosis OPG concentrations and breast cancer-specific mortality among ER+ breast cancer patients. Given a beneficial effect of the exogenous RANKL inhibitor denosumab for disease-free survival was previously shown [8], an inverse association was hypothesized for OPG, as it is an endogenous RANKL inhibitor. While this is challenging to reconcile, perhaps the most plausible explanation is through TRAIL. In addition to its role in blocking RANKL signaling, OPG inhibits TRAIL signaling [6]. However, this effect has predominantly been observed in experimental models of hormone receptor/triple negative breast cancer [6, 36]. We were unable to investigate OPG in triple negative breast cancer, as our study included insufficient numbers; 58 triple negative breast cancer cases and 12 deaths (9 of breast cancer). It merits noting that our study, as with most epidemiologic studies, used tumor ER status from the initial primary tumor; updated tumor ER status from recurrence or metastases was not available. Conversion from receptor positive to negative disease from primary tumor to recurrence [37] or distant metastases [38] has been described. For example, among 312 cases with systemic relapse, Lindstrom et al. reported 28.5% of cases ER+ in the primary tumor converted to ER- disease at relapse, whereas only 8.3% of patients converted from ER- to ER+ disease [37]. Therefore, it is likely that a proportion of the fatal ER+ cases in our study converted to ER- disease during disease progression.

Our analyses showed significant interaction between pre-diagnosis OPG and BMI, with an increased risk of death among ER+ breast cancer cases with a normal or underweight BMI at blood collection. We saw no apparent association in those with an overweight or obese baseline BMI. Experimental studies have shown that both ER activation and 17beta-estradiol treatment may downregulate OPG expression in the tumor [6]. It is possible that estrogens produced by adipose tissue in obese women [39] reduce OPG levels at the breast tissue level. However, we noted no correlation between circulating pre-diagnosis OPG and BMI in all cases (*r* = 0.02) or by menopausal status (r = <− 0.03), nor a correlation between OPG and estradiol in all cases (*r* = − 0.05) or by menopausal status (r = <− 0.06) in all cases. OPG concentrations did not differ by ever use of OCs in premenopausal women (p_dif_ 0.95) or PMH use at blood collection in postmenopausal women (p_dif_ 0.86).

We measured pre-diagnosis concentrations of sRANKL and OPG a median of 4.7 years before diagnosis. We have previously shown that OPG concentrations are reproducible over one and 14 years (*r* = 0.85 and *r* = 0.75 respectively) [9]. Reproducibility of sRANKL was lower (*r* = 0.60 and *r* = 0.38 over one and 14-years, respectively) [12]. This indicates that a single sRANKL measurement may not be representative of longer-term exposure, and may lead to attenuation of risk estimates. Nevertheless, within-person stability of sRANKL and OPG concentrations in the current study is higher than that of many sex steroid hormones. For example, in both pre-and postmenopausal women, within-person stability of estradiol, estrone, progesterone, and prolactin, assessed using intra-class correlations coefficients, have been reported to be under 0.45 both over one year and twenty years [40–42]. Further, the relatively high inter-batch CVs for OPG indicate measurement error may have led to non-differential misclassification and attenuation of risk estimates.

Pre-diagnosis concentrations of OPG and sRANKL may influence survival at a number of different stages in the disease process—for example, impacting initiation of a more vs. less aggressive tumor subtype and/or impacting tumor progression and/or influencing survival post-diagnosis (e.g. by interacting with treatment). It is plausible that concentrations at diagnosis are a more informative measure for breast cancer mortality. Literature on circulating concentrations of RANKL and OPG before and after onset of breast cancer is limited. In one study comparing serum samples taken before and after breast cancer diagnosis in 19 women, sRANKL concentrations were lower and OPG concentrations were higher after breast cancer diagnosis [10]. It would be of interest to further investigate these differences in larger studies that can account for e.g. tumor characteristics. We have recently shown a positive association between OPG concentrations and risk of ER- breast cancer (tertile 3 vs. 1 RR = 1.93 [95% CI 1.24–3.02]; p_trend_ = 0.03) [9], whereas higher sRANKL concentrations were associated with risk of ER+ disease (quintile 5 vs. 1 RR 1.28 [95%CI 1.01–1.63]; p_trend_ 0.20) [12]. Results from our current study indicate circulating concentrations of OPG and sRANKL may impact cancer risk and mortality differently, though further studies are required to more fully understand the underlying mechanisms.

## Conclusions

Higher pre-diagnosis endogenous concentrations of the decoy receptor for RANKL, OPG, appear to increase risk of death after a breast cancer diagnosis especially in those diagnosed with ER+ disease. Further investigations in well-defined patient cohorts are needed to confirm these results, and to clarify whether circulating OPG may be relevant for breast cancer prognosis.

## Additional file

Additional file 1: **Table S1.** Circulating concentrations of OPG and risk of death following a breast cancer diagnosis, by ER subtype and stratified by BMI at blood collection. **Table S2** The sRANKL/OPG ratio and risk of death following a breast cancer diagnosis, by ER subtype. **Table S3** sRANKL/OPG cross-classification and risk of death following a breast cancer diagnosis, by ER subtype. (DOCX 43 kb)

## Abbreviations

BMI: Body mass index
CI: 95% confidence interval
DKFZ: German Cancer Research Center
EPIC: European Prospective Investigation into Cancer and Nutrition
ER: Estrogen receptor
HR: Hazard ratio
IARC: International Agency for Research on Cancer
LLOD: Lower limit of detection
OPG: Osteoprotegerin
PMH: Postmenopausal hormone
PR: Progesterone receptor
RANK: Receptor activator of nuclear factor kappa-B
RANKL: RANK-ligand
sRANKL: soluble RANKL
TNF: Tumor necrosis superfamily
TRAIL: TNF related apoptosis inducing ligand
