# Supplementary material for: Receptor activator of nuclear factor kB ligand, osteoprotegerin, and risk of death following a breast cancer diagnosis: results from the EPIC cohort
Source: BMC Cancer. 2018 Oct 22;18:1010. doi: 10.1186/s12885-018-4887-3 (PMC6196438; doi:10.1186/s12885-018-4887-3)
Supplement: Supplementary file 1 — Table S1. Circulating concentrations of OPG and risk of death following a breast cancer diagnosis, by ER subtype and stratified by BMI at blood collection. Table S2 The sRANKL/OPG ratio and risk of death following a breast cancer diagnosis, by ER subtype. Table S3 sRANKL/OPG cross-classification and risk of death following a breast cancer diagnosis, by ER subtype. (DOCX 43 kb) [file 12885_2018_4887_MOESM1_ESM.docx]

| **Table S1. Circulating concentrations of OPG and risk of death following a breast cancer diagnosis, by ER subtype and stratified by BMI at blood collection.** | | | | | | | | |
| --- | --- | --- | --- | --- | --- | --- | --- | --- |
|  | Quintiles | | | | |  |  |  |
|  | 1 | 2 | 3 | 4 | 5 |  |  |  |
| *Cut points (pmol/L)* | ≤ 7.70 | 7.80 - 9.18 | 9.18 - 10.54 | 10.54 - 12.38 | > 12.38 | p_trend_^a^ | HR_log2_ | p_het_^b^ |
| **Breast cancer-specific death** | | | | | | |  |  |
| **BMI at blood collection <25kg/m^2^** | | | | | | |  |  |
| All breast cancer cases | | | | | | |  |  |
| *Died/total* | 27/241 | 21/224 | 19/218 | 24/221 | 28/215 |  | 119/1119 |  |
| *HR (95% CI)* | Ref. | 0.94 (0.52, 1.70) | 1.03 (0.56, 1.89) | 1.31 (0.73, 2.36) | 1.87 (1.04, 3.39) | 0.06 | 1.60 (0.99, 2.62) |  |
| *sRANKL adjusted*  *HR (95% CI)^c^* | Ref. | 0.96 (0.53, 1.74) | 1.05 (0.57, 1.95) | 1.29 (0.71, 2.35) | 1.94 (1.05, 3.57) | 0.06 | 1.62 (0.92, 1.17) |  |
|  |  |  |  |  |  |  |  |  |
| ER+ breast cancer cases | | | | | | |  |  |
| *Died/total* | 14/183 | 13/177 | 10/172 | 17/180 | 20/176 |  | 74/888 |  |
| *HR (95% CI)* | Ref. | 1.15 (0.52, 2.51) | 1.10 (0.47, 2.55) | 1.86 (0.88, 3.92) | 2.80 (1.30, 6.02) | 0.01 | 2.27 (1.22, 4.24) |  |
| *sRANKL adjusted*  *HR (95% CI)^c^* | Ref. | 1.23 (0.56, 2.71) | 1.18 (0.51, 2.76) | 1.92 (0.89, 4.16) | 3.19 (1.46, 6.97) | 0.005 | 2.53 (1.33, 4.83) |  |
|  |  |  |  |  |  |  |  |  |
| ER- breast cancer cases | | | | | | |  |  |
| *Died/total* | 13/58 | 8/47 | 9/46 | 7/41 | 8/39 |  | 45/231 |  |
| *HR (95% CI)* | Ref. | 0.87 (0.33, 2.26) | 0.98 (0.37, 2.59) | 0.84 (0.31, 2.30) | 1.12 (0.38, 3.29) | 0.97 | 0.99 (0.44, 2.21) |  |
| *sRANKL adjusted*  *HR (95% CI)^c^* | Ref. | 0.82 (0.31, 2.18) | 0.93 (0.33, 2.57) | 0.74 (0.26, 2.13) | 0.92 (0.28, 3.00) | 0.68 | 0.83 (0.34, 2.03) |  |
|  |  |  |  |  |  |  |  |  |
| **BMI at blood collection ≥25kg/m^2^** | | | | | | |  |  |
| All breast cancer cases | | | | | | |  |  |
| *Died/total* | 24/161 | 27/177 | 25/183 | 31/180 | 24/186 |  | 131/887 |  |
| *HR (95% CI)* | Ref. | 1.30 (0.72, 2.32) | 0.99 (0.53, 1.83) | 1.35 (0.75, 2.45) | 0.99 (0.52, 1.88) | 0.83 | 1.05 (0.67, 1.65) | 0.18 |
| *sRANKL adjusted*  *HR (95% CI)^c^* | Ref. | 1.32 (0.73, 2.40) | 1.05 (0.56, 1.98) | 1.34 (0.72, 2.50) | 1.06 (0.54, 2.08) | 0.78 | 1.07 (0.66, 1.72) |  |
|  |  |  |  |  |  |  |  |  |
| ER+ breast cancer cases | | | | | | |  |  |
| *Died/total* | 17/135 | 22/144 | 17/150 | 18/148 | 17/155 |  | 91/732 |  |
| *HR (95% CI)* | Ref. | 1.39 (0.71, 2.70) | 1.06 (0.50, 2.22) | 1.16 (0.56, 2.44) | 1.06 (0.49, 2.34) | 0.75 | 0.91 (0.50, 1.64) | 0.03 |
| *sRANKL adjusted*  *HR (95% CI)^c^* | Ref. | 1.51 (0.76, 3.00) | 1.17 (0.55, 2.50) | 1.18 (0.55, 0.55) | 1.16 (0.51, 0.64) | 0.72 | 0.90 (0.48, 1.66) |  |
|  |  |  |  |  |  |  |  |  |
| ER- breast cancer cases | | | | | | |  |  |
| *Died/total* | 7/26 | 5/33 | 8/33 | 13/32 | 7/31 |  | 40/155 |  |
| *HR (95% CI)* | Ref. | 1.21 (0.32, 4.50) | 0.97 (0.30, 3.16) | 2.34 (0.76, 7.20) | 0.98 (0.30, 3.21) | 0.36 | 1.43 (0.66, 3.10) | 0.36 |
| *sRANKL adjusted*  *HR (95% CI)^c^* | Ref. | 0.96 (0.24, 3.88) | 0.98 (0.28, 3.45) | 2.16 (0.66, 7.06) | 0.96 (0.27, 3.40) | 0.30 | 1.56 (0.67, 3.63) |  |
| **Death of any cause** | | | | | | |  |  |
| **BMI at blood collection <25kg/m^2^** | | | | | | |  |  |
| All breast cancer cases | | | | | | |  |  |
| *Died/total* | 39/241 | 36/224 | 36/218 | 43/221 | 54/215 |  | 208/1119 |  |
| *HR (95% CI)* | Ref. | 0.98 (0.62, 1.57) | 1.06 (0.66, 1.71) | 1.26 (0.79, 1.99) | 1.55 (0.97, 2.46) | 0.01 | 1.66 (1.15, 2.41) |  |
| *sRANKL adjusted*  *HR (95% CI)^c^* | Ref. | 0.99 (0.62, 1.59) | 1.00 (0.62, 1.63) | 1.17 (0.73, 1.88) | 1.36 (0.83, 2.22) | 0.05 | 1.49 (1.00, 2.20) |  |
|  |  |  |  |  |  |  |  |  |
| ER+ breast cancer cases | | | | | | |  |  |
| *Died/total* | 22/183 | 26/177 | 23/172 | 35/180 | 43/176 |  | 149/888 |  |
| *HR (95% CI)* | Ref. | 1.23 (0.68, 2.21) | 1.21 (0.66, 2.24) | 1.72 (0.98, 3.02) | 1.98 (1.11, 3.51) | 0.002 | 1.98 (1.28, 3.06) |  |
| *sRANKL adjusted*  *HR (95% CI)^c^* | Ref. | 1.23 (0.68, 2.21) | 1.17 (0.63, 2.16) | 1.63 (0.92, 0.89) | 1.82 (1.00, 3.31) | 0.008 | 1.86 (1.18, 2.95) |  |
|  |  |  |  |  |  |  |  |  |
| ER- breast cancer cases | | | | | | |  |  |
| *Died/total* | 17/58 | 10/47 | 13/46 | 8/41 | 11/39 |  | 59/231 |  |
| *HR (95% CI)* | Ref. | 0.69 (0.29, 1.63) | 0.95 (0.42, 2.17) | 0.65 (0.26, 1.64) | 1.18 (0.48, 2.91) | 0.66 | 1.18 (0.57, 2.42) |  |
| *sRANKL adjusted*  *HR (95% CI)^c^* | Ref. | 0.66 (0.28, 1.58) | 0.79 (0.33, 1.91) | 0.53 (0.20, 1.39) | 0.78 (0.27, 2.22) | 0.71 | 0.85 (0.38, 1.94) |  |
|  |  |  |  |  |  |  |  |  |
| **BMI at blood collection ≥25kg/m^2^** | | | | | | |  |  |
| All breast cancer cases | | | | | | |  |  |
| *Died/total* | 38/161 | 35/177 | 35/183 | 50/180 | 55/186 |  | 213/887 |  |
| *HR (95% CI)* | Ref. | 0.85 (0.53, 1.38) | 0.67 (0.40, 1.09) | 0.94 (0.59, 1.49) | 0.94 (0.58, 1.51) | 0.66 | 1.09 (0.75, 1.57) | 0.03 |
| *sRANKL adjusted*  *HR (95% CI)^c^* | Ref. | 0.88 (0.54, 1.42) | 0.69 (0.42, 0.15) | 0.92 (0.57, 1.49) | 0.99 (0.60, 1.62) | 0.64 | 1.10 (0.74, 1.62) |  |
|  |  |  |  |  |  |  |  |  |
| ER+ breast cancer cases | | | | | | |  |  |
| *Died/total* | 31/135 | 28/144 | 24/150 | 36/148 | 42/155 |  | 161/732 |  |
| *HR (95% CI)* | Ref. | 0.81 (0.48, 1.37) | 0.62 (0.35, 1.10) | 0.85 (0.50, 1.45) | 0.92 (0.53, 1.58) | 0.95 | 1.02 (0.65, 1.59) | 0.003 |
| *sRANKL adjusted*  *HR (95% CI)^c^* | Ref. | 0.88 (0.51, 1.50) | 0.66 (0.37, 1.18) | 0.86 (0.50, 1.49) | 0.99 (0.56, 1.74) | 0.93 | 1.02 (0.64, 1.63) |  |
|  |  |  |  |  |  |  |  |  |
| ER- breast cancer cases | | | | | | |  |  |
| *Died/total* | 7/26 | 2/33 | 11/33 | 14/32 | 13/31 |  | 52/155 |  |
| *HR (95% CI)* | Ref. | 1.56 (0.47, 5.15) | 1.21 (0.40, 3.70) | 1.99 (0.68, 5.83) | 1.38 (0.47, 3.99) | 0.29 | 1.45 (0.73, 2.89) | 0.39 |
| *sRANKL adjusted*  *HR (95% CI)^c^* | Ref. | 1.30 (0.37, 4.05) | 1.13 (0.35, 3.67) | 1.71 (0.56, 5.22) | 1.28 (0.42, 3.92) | 0.31 | 1.47 (0.70, 3.10) |  |
| *All models adjusted for breast cancer stage (localized, non-localized, missing), BMI (kg/m^2^), age at blood collection (years), and age groups at menarche (≤11, 12, 13 and missing, 14, ≥15 years), menopause (premenopausal ≤48, 49-51, ≥52 years, missing), and first full term pregnancy (nulliparous, <25 years, ≥25 years and missing); stratifying by age groups at diagnosis (5 year groups) and models in all cases by ER status of the tumor.*  *^a^ p_trend_ based on log2-transformed OPG concentrations;* ^b^ *p_heterogeneity_ comparing model without to model with interaction term for OPG and BMI (</≥ 25kg/m^2^) using log likelihood ratio tests; ^c^ additionally adjusting for sRANKL concentrations (42 observations missing sRANKL concentrations). Analyses in those with a BMI <25kg/m^2^ include 118 breast cancer deaths and 204 deaths of any cause, analyses in those with a BMI ≥25kg/m^2^ include 127 breast cancer deaths and 208 deaths of any cause.* | | | | | | | | |

| **Table S2. The sRANKL/OPG ratio and risk of death following a breast cancer diagnosis, by ER subtype.** | | | | | | | | | |
| --- | --- | --- | --- | --- | --- | --- | --- | --- | --- |
|  | Quintiles | | | | | |  |  |  |
|  | 1 | | 2 | 3 | 4 | 5 |  |  |  |
| *Cut points* | ≤ 0.0036 | | 0.0036 – 0.0083 | 0.0083 – 0.0143 | 0.0143 – 0.0238 | > 0.0238 | p_trend_^a^ | HR_log2_ | p_het_^b^ |
| **Breast cancer-specific survival** | | | | | | | |  |  |
| All breast cancer cases | | | | | | | |  |  |
| *Died/total* | | 49/393 | 49/393 | 41/393 | 52/393 | 54/393 |  | 245/1965 |  |
| *HR (95% CI)* | | Ref. | 0.92 (0.61, 1.38) | 0.75 (0.49, 1.14) | 0.98 (0.66, 1.47) | 0.89 (0.59, 1.36) | 0.65 | 0.98 (0.92, 1.06) | 0.49 |
|  | |  |  |  |  |  |  |  |  |
| ER+ breast cancer cases | | | | | | | |  |  |
| *Died/total* | | 31/318 | 34/325 | 24/311 | 36/311 | 37/323 |  | 162/1588 |  |
| *HR (95% CI)* | | Ref. | 0.98 (0.59, 1.61) | 0.75 (0.44, 1.30) | 1.13 (0.69, 1.86) | 0.88 (0.53, 1.48) | 0.88 | 0.99 (0.91, 1.09) |  |
|  | |  |  |  |  |  |  |  |  |
| ER- breast cancer cases | | | | | | | |  |  |
| *Died/total* | | 18/75 | 15/68 | 17/82 | 17/82 | 16/70 |  | 83/377 |  |
| *HR (95% CI)* | | Ref. | 0.86 (0.41, 1.79) | 0.76 (0.38, 1.54) | 0.81 (0.40, 1.65) | 0.87 (0.42, 1.81) | 0.63 | 0.97 (0.86, 1.10) |  |
| **Death of any cause** | | | | | | | |  |  |
| All breast cancer cases | | | | | | | |  |  |
| *Died/total* | | 104/393 | 86/393 | 66/393 | 77/393 | 79/393 |  | 412/1965 |  |
| *HR (95% CI)* | | Ref. | 0.80 (0.59, 1.07) | 0.63 (0.46, 0.86) | 0.77 (0.57, 1.03) | 0.82 (0.59, 1.12) | 0.05 | 0.95 (0.90, 1.00) | 0.35 |
|  | |  |  |  |  |  |  |  |  |
| ER+ breast cancer cases | | | | | | | |  |  |
| *Died/total* | | 76/318 | 68/325 | 43/311 | 57/311 | 60/323 |  | 304/1588 |  |
| *HR (95% CI)* | | Ref. | 0.85 (0.60, 1.19) | 0.59 (0.41, 0.87) | 0.79 (0.56, 1.13) | 0.83 (0.58, 1.20) | 0.16 | 0.96 (0.90, 1.02) |  |
|  | |  |  |  |  |  |  |  |  |
| ER- breast cancer cases | | | | | | | |  |  |
| *Died/total* | | 28/75 | 18/68 | 23/82 | 21/82 | 18/70 |  | 108/377 |  |
| *HR (95% CI)* | | Ref. | 0.69 (0.36, 1.30) | 0.66 (0.36, 1.21) | 0.70 (0.38, 1.29) | 0.71 (0.40, 1.35) | 0.13 | 0.92 (0.83, 1.03) |  |
| *All models adjusted for breast cancer stage (localized, non-localized, missing), BMI (kg/m^2^), age at blood collection (years), and age groups at menarche (≤11, 12, 13 and missing, 14, ≥15 years), menopause (premenopausal ≤48, 49-51, ≥52 years, missing), and first full term pregnancy (nulliparous, <25 years, ≥25 years and missing); stratifying by age groups at diagnosis (5 year groups) and models in all cases by ER status of the tumor.*  *^a^ p_trend_ based on log2-transformed sRANKL/OPG ratio;* ^b^ *p_heterogeneity_ comparing model without to model with interaction term for sRANKL/OPG ratio and ER status using log likelihood ratio tests.* | | | | | | | | | |

| **Table S3. sRANKL/OPG cross-classification and risk of death following a breast cancer diagnosis, by ER subtype.** | | | | |
| --- | --- | --- | --- | --- |
|  | Category | | | |
| *sRANKL/OPG* | </> median | </< median | >/> median | >/< median |
| **Breast cancer specific death** | | | | |
| All breast cancer cases | | | | |
| *Died/total* | 71/582 | 40/364 | 54/401 | 80/618 |
| *HR (95% CI)* | Ref. | 0.80 (0.54, 1.19) | 1.13 (0.79, 1.62) | 0.93 (0.66, 1.31) |
|  |  |  |  |  |
| ER+ breast cancer cases | | | | |
| *Died/total* | 49/480 | 23/281 | 34/332 | 56/495 |
| *HR (95% CI)* | Ref. | 0.69 (0.42, 1.15) | 1.02 (0.65, 1.59) | 0.92 (0.61, 1.40) |
|  |  |  |  |  |
| ER- breast cancer cases | | | | |
| *Died/total* | 22/102 | 17/83 | 20/69 | 24/123 |
| *HR (95% CI)* | Ref. | 0.86 (0.44, 1.68) | 1.46 (0.77, 2.77) | 0.88 (0.47, 1.64) |
| **Death of any cause** | | | | |
| All breast cancer cases | | | | |
| *Died/total* | 146/582 | 65/364 | 86/401 | 115/618 |
| *HR (95% CI)* | Ref. | 0.76 (0.56, 1.03) | 0.93 (0.71, 1.22) | 0.83 (0.64, 1.08) |
|  |  |  |  |  |
| ER+ breast cancer cases | | | | |
| *Died/total* | 115/480 | 40/281 | 62/332 | 87/495 |
| *HR (95% CI)* | Ref. | 0.63 (0.44, 0.92) | 0.83 (0.61, 1.14) | 0.82 (0.61, 1.11) |
|  |  |  |  |  |
| ER- breast cancer cases | | | | |
| *Died/total* | 31/102 | 25/83 | 24/69 | 28/123 |
| *HR (95% CI)* | Ref. | 0.99 (0.56, 1.74) | 1.31 (0.75, 2.30) | 0.82 (0.47, 1.42) |
| *All models adjusted for breast cancer stage (localized, non-localized, missing), BMI (kg/m^2^), age at blood collection (years), and age groups at menarche (≤11, 12, 13 and missing, 14, ≥15 years), menopause (premenopausal ≤48, 49-51, ≥52 years, missing), and first full term pregnancy (nulliparous, <25 years, ≥25 years and missing); stratifying by age groups at diagnosis (5 year groups) and models in all cases by ER status of the tumor.* | | | | |
